# A novel DAG-dependent mechanism links PKCa and Cyclin B1 regulating cell cycle progression

**DOI:** 10.18632/oncotarget.2578

**Published:** 2014-10-24

**Authors:** Alessandro Poli, Giulia Ramazzotti, Alessandro Matteucci, Lucia Manzoli, Annalisa Lonetti, Pann-Ghill Suh, James A. McCubrey, Lucio Cocco

**Affiliations:** ^1^ Cell Signaling Laboratory, Department of Biomedical Sciences, University of Bologna, Bologna, BO 40126, Italy; ^2^ CNR-National Research Council of Italy, Institute of Molecular Genetics, Bologna, BO 40126, Italy; ^3^ School of Nano-Biotechnology and Chemical Engineering, Ulsan National Institute of Science and Technology, Ulsan, Republic of Korea; ^4^ Department of Microbiology and Immunology, Brody School of Medicine, East Carolina University, Greenville, NC, USA

**Keywords:** PKC, Cyclin, Cell Cycle, PLC, DAG, nuclei

## Abstract

Through the years, different studies showed the involvement of Protein Kinase C (PKC) in cell cycle control, in particular during G1/S transition. Little is known about their role at G2/M checkpoint. In this study, using K562 human erythroleukemia cell line, we found a novel and specific mechanism through which the conventional isoform PKC⍺ positively affects Cyclin B1 modulating G2/M progression of cell cycle. Since the kinase activity of this PKC isoform was not necessary in this process, we demonstrated that PKC⍺, physically interacting with Cyclin B1, avoided its degradation and stimulated its nuclear import at mitosis. Moreover, the process resulted to be strictly connected with the increase in nuclear diacylglycerol levels (DAG) at G2/M checkpoint, due to the activity of nuclear Phospholipase C β1 (PLCβ1), the only PLC isoform mainly localized in the nucleus of K562 cells. Taken together, our findings indicated a novel DAG dependent mechanism able to regulate the G2/M progression of the cell cycle.

## INTRODUCTION

Protein kinase C (PKC) is a family of serine/threonine kinases involved in different biological functions [1–3]. Ten PKCs are present in mammalian cells and are divided in three classes based on their structure domains and activation [1–3]. Indeed, activation of conventional PKCs (PKC⍺, βI, βII and γ) requires the lipid second messengers diacylglycerol (DAG) and Ca^2+^, while novel isozymes (PKC δ, ε, θ and η) need only DAG. On the contrary, the atypical class (PKCζ and λ/ι) is not sensible to any of them, and its activation is due to protein-protein interactions [1–3]. Our knowledge about the involvement of these enzymes in cell cycle regulation is very wide at the moment and, through the years, it became clear that these effects are linked to the different contexts where they take place [2–4]. As a matter of fact, many studies reported roles for PKCs in cell cycle both as anti-proliferative and growth-stimulatory enzymes [2–5]. Modulation of cell proliferation by PKCs is characterized by high complexity, effecting different molecules involved in the control of the cell cycle including cyclins, cyclin-dependent kinases (Cdk), Cip/Kip inhibitors and Lamins [2, 4–8]. However, several evidences indicated Cip/Kip inhibitors and D-type cyclins as the most frequent targets for PKCs. Indeed, many studies described the involvement of PKCs in G1/S transition regulating Cyclin D1, p21/Cip1 or p27/Kip1 expressions in different cell lines [2, 4, 8–11]. Recently, we found that PKC⍺ was necessary in PLCβ1 mediated regulation of Cyclin D3 and cell proliferation in human erythroleukemia cells [12, 13]. On the other hand, little is known about the role of PKCs at G2/M phase [2, 4]. Different studies showed their peculiar ability to partially translocate into the nuclei influencing this phase of the cell cycle. In particular, nuclear import of PKCs was correlated to the increase of nuclear diacylglycerol (DAG) before mitosis [6] [14] [15–18]. These findings were supported by Fiume et. al, who demonstrated that PKC⍺, once in the nuclei, could phosphorylate Lamin B1 stimulating lamin dissociation and G2/M progression [19]. In this study, investigating other possible roles for PKCs at G2/M phase, we found that Cyclin B1 can positively be modulated by PKC⍺. As widely described in literature, the entry of eukaryotic cells into mitosis is due to the activation of cyclin dependent kinase 1 (Cdk1), which complexes with its regulatory subunit Cyclin B1 to form the mitosis-promoting factor (MPF) [21–28]. MPF remains inactive until Cdk1 is phosphorylated at Thr161 by Cdk activating kinase (CAK) and de-phosphorylated by Cdc25c at Thr14/Thr15 [20–28]. In addition, Cyclin B1 is phosphorylated by Cdk1 and Polo-like kinase 1 (PLK1) in its cytoplasmic retention signal (CRS) domain, which regulates its nuclear translocation at late prophase [21–28]. This nuclear accumulation has been highly studied and described, but remains not completely understood for the lack of a canonical nuclear localization signal (NLS) in Cyclin B1 structure, usually necessary for nuclear import through the karyopherins system [21–29]. However, once in the nuclei, Cyclin B1/Cdk1 complex phosphorylates a wide number of substrates driving the cells into mitosis [20–28]. Finally, at the end of the mitotic process, Cyclin B1 starts to be degraded by the APC/C complex and Cdk1 undergoes inactivation leading cells to mitotic exit and cytokinesis [21–32]. Here, we describe, for the first time, a DAG dependent mechanism linking PKC⍺ to Cyclin B1 at G2/M checkpoint. Indeed, investigating whether PKCs could affect G2/M progression in K562 cell line, we found that Cyclin B1 was positively modulated by PKC⍺. This event was independent of the kinase activity of the enzyme. Moreover, PKC⍺ resulted to physically interact with Cyclin B1 during cell cycle progression, avoiding its degradation and promoting its nuclear accumulation. Finally, we observed how DAG accumulation in nucleus, due to the activity of nuclear PLCβ1, could modulate Cyclin B1 and PKC⍺ nuclear translocation at G2/M checkpoint.

## RESULTS

### PKCs affect Cyclin B1 levels in K562 cells

**Figure 1:**
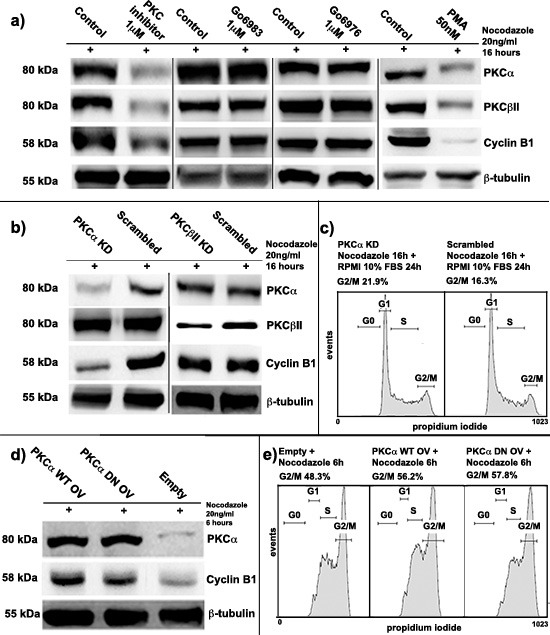
Protein kinase C ⍺ (PKC⍺) involvement in Cyclin B1 regulation **(a)** K562 cells were treated with three different PKC inhibitors (PKC inhibitor, Go6983, Go6976) and with phorbol-12-myristate-13-acetate (PMA). Cells were synchronized at G2/M by Nocodazole. PKCs decrease led to a severe downregulation of Cyclin B1 expression if compared to the controls (Control). **(b)** PKC⍺ and PKCβII were silenced for 48 hours (PKC⍺ KD/PKCβII KD). Cells were synchronized at G2/M using Nocodazole. A scrambled siRNA was used as control (Scrambled). Only PKC⍺ silencing drove to Cyclin B1 downmodulation. The silencing was performed using different siRNAs to avoid any possible off-target effects. **(c)** Cells, blocked at G2/M, were seeded again in complete RPMI 10% FBS. 24 hours later, cell cycle analysis was performed indicating an accumulation at G2/M in PKC⍺ knock-down conditions. **(d)** PKC⍺ was overexpressed using a Wild Type (PKC⍺ OV WT) or a Dominant Negative (PKC⍺ OV DN) vector. Cells were stimulated to accumulate at G2/M using Nocodazole for 6 hours. Empty vector was used as control (Empty). Overexpression of PKC⍺ WT and DN lead to an upmodulation of Cyclin B1. **(e)** Cells were treated using Nocodazole as in d) and cell cycle analysis was performed indicating an higher accumulation at G2/M of cells overexpressing PKC⍺.

In order to find whether PKCs could target Cyclin B1 expression during cell cycle of K562 cell line, we treated cells with three different PKC inhibitors at a final concentration of 1μM: Go6983, Go6976 and 3-(1-(3-imidazol-1-ylpropyl)-1H-indol-3-yl)-4-anilino-1H-pyrrole-2,5-dione anilinomonoindolylmaleimide (from here simply PKC inhibitor) [1, 2, 19] [33, 34]. Next, we synchronized the cells at G2/M checkpoint using Nocodazole (20ng/ml) for 16 hours to avoid any cell cycle-dependent modulation of Cyclin B1 expression. Notably, immunoblot analyses showed an important decrease of Cyclin B1 levels only in cells treated with PKC inhibitor. The effect appeared to be concomitant with the high reduction of PKC⍺ and PKCβII, the only two conventional PKC isoforms expressed in our model [12, 35, 36]. This down-modulation of the level of PKCs was not linked to their reduced gene expression (Supplementary Figure 1) but, probably, to side effects on other enzymes involved in PKC regulation. On the contrary, using Go6976 and Go6983, which inhibited only PKC activity [33], we did not observe any appreciable change in Cyclin B1 expression. In addition, we stimulated PKC signalling using Phorbol-12-Myristate-13-Acetate (PMA) at a final concentration of 50nM for 16 hours. Interestingly, synchronized K562 cells at G2/M checkpoint showed an important decrease of Cyclin B1 parallel to PKC⍺ and PKCβII degradation. PKCs degradation was probably due to their hyper-activation by PMA [35–37], Cyclin B1 down-modulation seemed to be related to the decrease of PKCs in the cells as well as during treatments with PKC inhibitor (Figure 1A).

### PKC⍺ is the conventional isoform that modulates Cyclin B1 affecting cell cycle progression

In order to understand which of the two conventional PKC isoforms was responsible of the phenomenon described above, we transiently silenced both of them using RNA interference techniques (PKC⍺ KD/PKCβII KD). More than one siRNA was used for the experiments in order to exclude any off-target effects and no changes in the results were found (see materials and methods). A Scrambled siRNA was used as control (Scrambled). 24 hours after transfection, using Nocodazole, we synchronized the cells at G2/M checkpoint. Here, we observed a strong down-regulation of Cyclin B1 only in cells characterized by PKC⍺ knock-down. PKCβII did not affect it at all (Figure 1B). Next, to confirm that this modulation was due to a specific effect of PKC⍺ on Cyclin B1, we screened other proteins related to G2/M progression. None of these molecules was affected (Supplementary Figure 2). In addition, we also investigated if PKC⍺ could be responsible for Cyclin B1 phosphorylations, including Ser133 and Ser147, but it was not (Supplementary Figure 2). However, we found that this specific modulation of Cyclin B1 could affect cell cycle progression. In order to investigate these effects, we silenced PKC⍺ and we synchronized the cells at G2/M. Then, we removed Nocodazole, we washed the cells in PBS and we seeded them again in complete RPMI 10% FBS. 24 hours later, cell cycle analyses indicated that PKC⍺ silencing drove the cells to a higher G2/M accumulation compared to the controls. This could be related to the lack of Cyclin B1 caused by PKC⍺ silencing. Indeed, accordingly to literature, a high decrease of Cyclin B1 could interfere with the transition through the G2/M phase of cell cycle [38]. Moreover, the same experiments were performed using PKC inhibitor, whose effects on cell cycle were stronger but very similar to PKC⍺ silencing (Supplementary Figure 3). Finally, we analyzed proliferation of K562 cell line. We silenced PKC⍺ and, 24 hours later, we synchronized the cells using Nocodazole. Then we removed the G2/M block seeding the transfected cells in complete RPMI 10% FBS at a cell density of 10 × 10^5^/ml. Experiments of cell counting, performed for 72 hours, showed that PKC⍺ silencing did slow-down cell proliferation of K562 cells compared to the controls. The same effects were noticed using PKC inhibitor (Supplementary Figure 4).

### PKC⍺ activity was not necessary for Cyclin B1 modulation

Since PKC⍺ positively modulated Cyclin B1 levels in K562 cells, we decided to investigate if its kinase activity was necessary to this process. Then, we transiently overexpressed PKC⍺ using two different vectors, a Wild Type vector (PKC⍺ OV WT) and a Dominant Negative mutant (PKC⍺ OV DN). Empty vector (Empty) was used as control. 24 hours after the transfections, we added Nocodazole for 6 hours to drive the cells to G2/M. This partial synchronization was performed to stimulate Cyclin B1 accumulation and study if PKC⍺ overexpression could promote this process. Notably, in cells characterized by PKC⍺ WT or DN overexpression, we found the same increase of Cyclin B1 compared to the controls (Figure 1D). As a further proof that the kinase activity of PKC⍺ was not necessary in this process, we analyzed cell cycle of these samples. Overexpression of the two PKC vectors, WT and DN, led the cells to a quicker accumulation at G2/M than the controls. In addition, cell counting analyses demonstrated that up-modulation of PKC⍺ was able to increase K562 cell proliferation (Supplementary Figure 4). Taken together, these data suggested that the kinase activity of PKC⍺ was not necessary in Cyclin B1 modulation.

### Lack of PKC⍺ leads to a faster degradation of Cyclin B1 in K562 cell line

**Figure 2:**
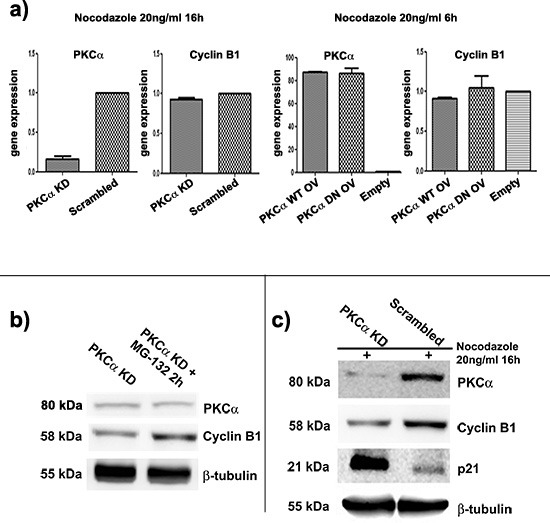
Lack of PKC⍺ drives to a faster degradation of Cyclin B1 **(a)** Gene expression analyses of Cyclin B1 in conditions of silencing or overexpression of PKC⍺. Cells were synchronized at G2/M. Cyclin B1 expression is not affected by modulation of PKC⍺. **(b)** PKC⍺ was silenced and, 48 hours later, transfected cells were divided in two aliquots: one used as control (PKC⍺ KD) and one treated with MG-132 for 2 hours (PKC⍺ KD + MG-132 2h) The treatment blocked Cyclin B1 degradation and led to its accumulation in the cells. No changes in PKC⍺ levels were detected. These evidences suggested that down-modulation of Cyclin B1 due to lack of PKC⍺ was ceased inhibiting the proteasome machinery. **(c)** PKC⍺ was silenced and cells synchronized at G2/M. Silencing of PKC⍺ (PKC⍺ KD) leads to an accumulation of p21 compared to the control (Scrambled). This effect is opposite to Cyclin B1 decrease. p21 was used as a marker of degradation for Cyclin B1.

In order to deeply understand how PKC⍺ could affect Cyclin B1, we decided to investigate at which level this modulation took place. We silenced or overexpressed PKC⍺ and we synchronized K562 cells as previously described. We performed gene expression analyses of Cyclin B1, but we did not find any changes in its expression levels (Figure 2A). These evidences suggested the existence of a post-transcriptional effect on Cyclin B1 by PKC⍺. Then, we focused our studies on Cyclin B1 degradation, implying a possible role for PKC⍺ as a scaffold molecule. As already reported [30, 31], Cyclin B1 undergoes degradation at the end of mitosis. Thus, we used a proteasome inhibitor, MG-132, to block protein degradation after G2/M release. Our findings showed that treating K562 cells with MG-132 up to two hours, at a final concentration of 15μM, blocked Cyclin B1 degradation driving to its accumulation in the cells (Supplementary Figure 5). Then, we silenced PKC⍺ and, 48 hours later, we divided transfected cells in two aliquots; one was treated with MG-132 for two hours, one was seeded in complete RPMI 1640 10% FBS and used as control. Notably, MG-132 treated cells showed a peculiar and significant increase of Cyclin B1 levels compared to the control. PKC⍺ expression remained silenced in both samples (Figure 2B). These evidences indicated that the presence of PKC⍺ inhibited the degradation of Cyclin B1 in K562 cells. In addition, as a positive control, we decided to investigate the Cip inhibitor p21 [39], already described to highly accumulate during Cyclin B1 degradation [40]. Interestingly, in cells transfected to silence PKC⍺, the decrease of Cyclin B1 was concomitant with an important up-modulation of p21/Cip1 levels (Figure 2C). Taken together, these data supported our theory, showing that lack of PKC⍺ drove to a faster degradation of Cyclin B1 in K562 cells.

### Cyclin B1 and PKC⍺ interact during cell cycle progression

**Figure 3:**
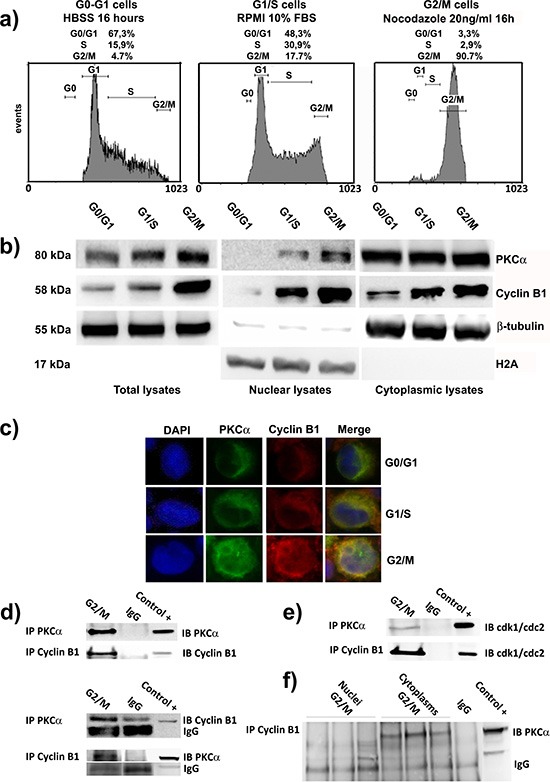
PKC⍺ and Cyclin B1 accumulate into the nuclei and interact at G2/M **(a)** Cells were synchronized in G0/G1 by HBSS (G0/G1), G1/S by complete RPMI 10% FBS (G1/S) and G2/M by Nocodazole (G2/M). **(b)** Immunoblot analysis of total, nuclear and cytoplasmic lysates of cells synchronized at different cell cycle phases was performed to investigat PKC⍺ and Cyclin B1 expression and localization during cell cycle. PKC⍺ and Cyclin B1 increase and translocate into the nuclei during cell cycle, peaking at G2/M checkpoint. **(c)** Cells were synchronized as a) and analyzed using immunocytochemistry to study PKC⍺ and Cyclin B1 localization. The two enzymes co-localize during cell cycle progression and accumulate into the nuclei in particular at G2/M transition. **(d)** Co-immunoprecipitations experiments in cells synchronized at G2/M (G2/M) indicated the possibility for PKC⍺ and Cyclin B1 to complex. As negative control non-specific IgG were used (IgG). 50μg total lysate of K562 were used as positive control (Control +). To show that the experiments were performed correctly, immunoblots of the immunoprecipitated proteins are provided. All the experiments were performed more than four times. **(e)** Cells were blocked at G2/M checkpoint and PKC⍺ or Cyclin B1 were immunoprecipitated. An antibody anti-cdk1/cdc2 was used to detect the capacity for both the proteins to interact with this kinase. **(f)** The interaction between Cyclin B1 and PKC⍺ takes place in the cytoplasms. Nuclei and cytoplasms of cells synchronized in G2/M were separated. Cyclin B1 was immunoprecipitated and PKC⍺ was detected via Western Blotting. The complex between the two enzymes was appreciable only in the cytoplasmic fractions of the cells. The experiment was repeated three times as shown in the figure.

In this series of experiments, we focused on the expression and localization of Cyclin B1 and PKC⍺ during cell cycle progression. Then, we treated our cells to promote their accumulation in the desired cell cycle phases. In particular, we starved K562 cells, using HBSS for 16 hours, to obtain a G0-G1 synchronization (G0-G1). G1/S phase was referred to proliferating cells in normal and complete RPMI 1640 10% FBS (G1/S). Finally, using Nocodazole, we obtained a G2/M block (G2/M) (Figure 3A). Firstly, immunoblot analyses showed a progressive increase of Cyclin B1 and PKC⍺ expression through the cell cycle peaking at G2/M. Next, separating nuclei of K562 cells from the cytoplasms, we investigated their localization along the different phases. Notably, PKC⍺ accumulated in the nucleus especially in G2/M cells as well as Cyclin B1 (Figure 3B). This similar co-localization was also confirmed by immunocytochemistry analyses (Figure 3C). Next, we decided to see if these two proteins could interact during cell cycle. After seeing that our immunoprecipitation experiments worked properly (Figure 3D), we synchronized the cells at G2/M checkpoint and we performed co-immunoprecipitation analyses to seek the possible formation of complexes between the two enzymes. Interestingly, our findings showed that PKC⍺ and Cyclin B1 could interact (Figure 3D). As in the negative control (IgG) of PKC⍺ immunoprecipitation appeared a partial non-specific band, probably due to cross-reaction between the antibody, we repeated the experiments more than 4 times. Moreover, interaction between the two proteins was detected also in G1/S cycling cells, which confirmed the capacity of PKC⍺ and Cyclin B1 to complex during cell cycle progression (Supplementary Figure 6). Next, in order to further investigate the existence of this complex, we again immunoprecipitated PKC⍺ and we found it was able to interact with cdk1/cdc2, the kinase that forms the MPF associating with Cyclin B1 [20–25], [26], [27, 28]. As positive control of the experiment, we immunoprecipitated Cyclin B1 finding it perfectly complexed with cdk1/cdc2 (Figure 3E). Finally, we repeated these experiments separating nuclei from cytoplasms of cells synchronized at G2/M and we found that Cyclin B1 and PKC⍺ were able to interact in the cytoplasmic fraction (Figure 3F). These data indicated that the interaction between the two proteins could be very important in the prevention of Cyclin B1 degradation during cell cycle progression.

### Cyclin B1 nuclear accumulation is modulated by the changes of PKC⍺ expression

**Figure 4:**
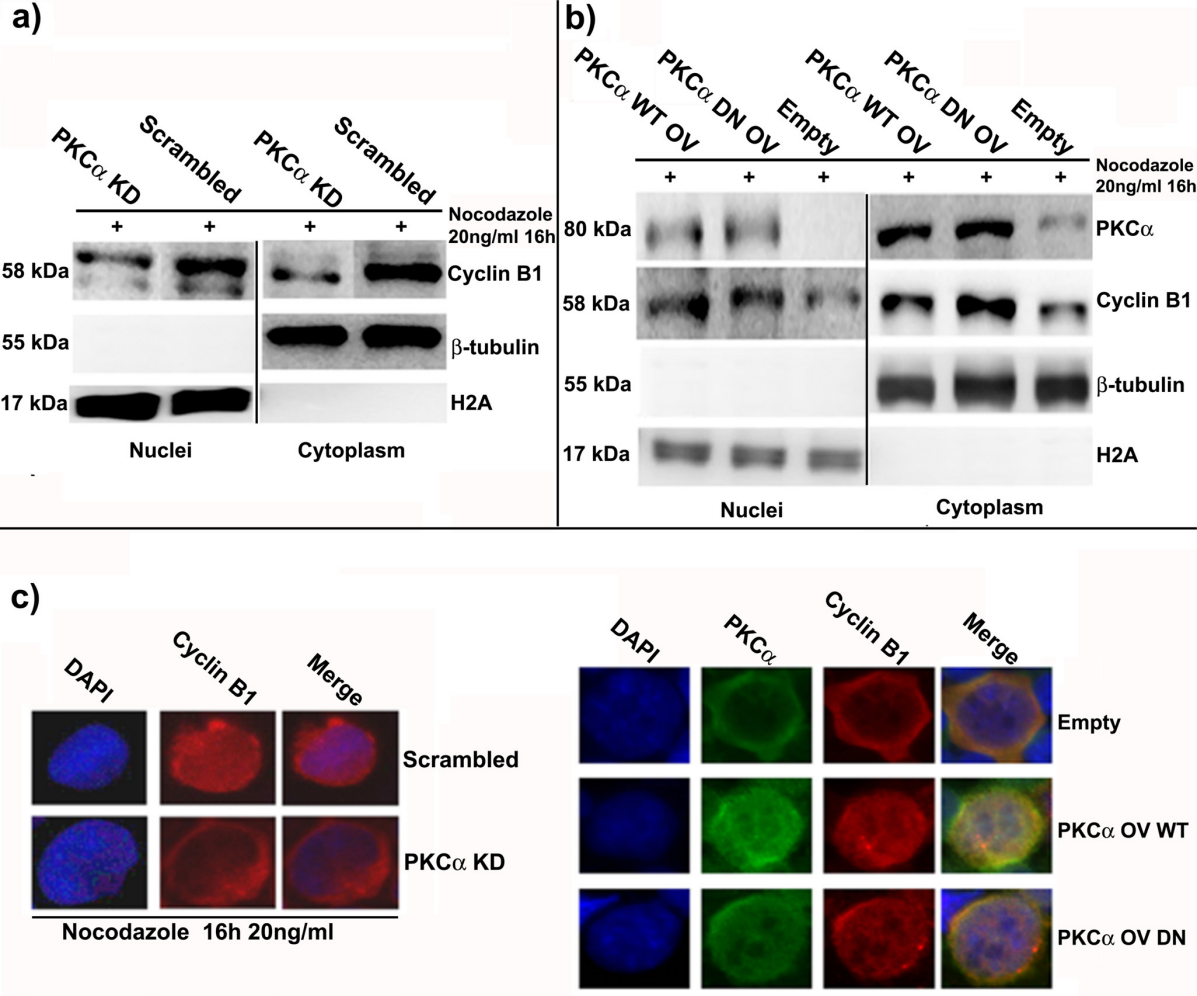
PKC⍺ silencing or overexpression modulate the amount of Cyclin B1 in the nucleus at G2/M checkpoint **(a)** PKC⍺ was silenced and, 48 hours later, cells were synchronized using Nocodazole. A Scrambled siRNA was used as control (Scrambled). Then, nuclei and cytoplasms were separated and Cyclin B1 localization was investigated. Cyclin B1 decreases both in the cytoplasms and in the nuclei. **(b)** PKC⍺ was overexpressed using WT and DN vectors and cells were treated with Nocodazole for 6 hours. An Empty vector (Empty) was used as control. Cyclin B1 increases both in the nuclei and the cytoplasms. **(c)** Cells were treated as reported in a) and b) and immunocytochemistry was performed to study the localization of Cyclin B1.

As reported above, PKC⍺ and Cyclin B1 co-localize and interact, in particular during G2/M progression. Both of these molecules can accumulate into the nucleus during this phase of the cell cycle, but how this mechanism works is still not well understood [24]. In these series of experiments, we showed that lack or increase of PKC⍺, and its consequent minor or major nuclear translocation at mitosis, affected Cyclin B1 nuclear import. We silenced or overexpressed PKC⍺ in cells treated with Nocodazole as previously described and we separated nuclei from cytoplasms. Silencing of PKC⍺ led to a decrease of Cyclin B1 levels in cytoplasms and in nuclei of K562 cells synchronized at G2/M checkpoint (Figure 4A). On the contrary, overexpression of PKC⍺ drove to a higher accumulation of Cyclin B1 both into the nuclear and cytoplasmic fractions (Figure 4B). All these data were confirmed by immunocytochemistry experiments and using PKC inhibitor (Figure 4C and Supplementary Figure 7A and 7B). Taken together, these evidences indicated that the more PKC⍺ translocates in the nucleus at G2/M checkpoint the more nuclear accumulation of Cyclin B1 takes place in K562 cell line.

### Nuclear DAG increase is required for PKC⍺ and Cyclin B1 translocation into the nucleus

**Figure 5:**
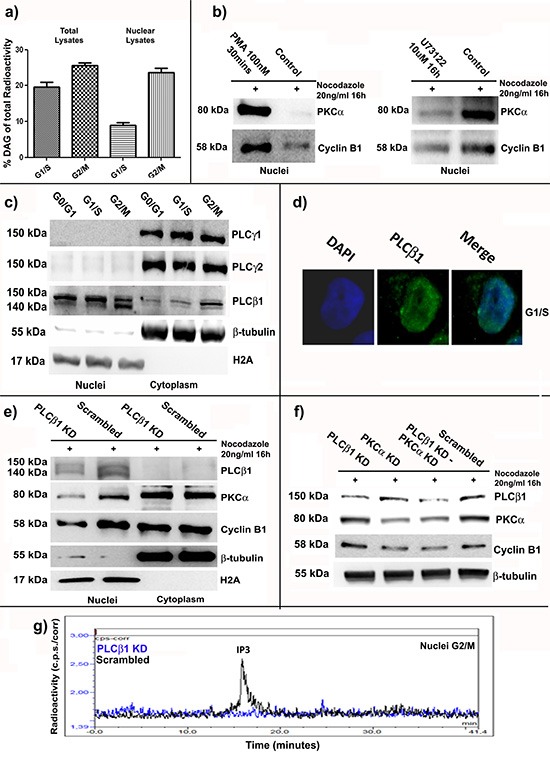
PLCβ1 related nuclear DAG increase at G2/M stimulates nuclear translocation of PKC⍺ and Cyclin B1 **(a)** Cells were synchronized at G1/S and G2/M and nuclear or total samples were treated to extract lipids. The percentage of radioactivity corresponding to DAG amount was investigated. Nuclear DAG increased at G2/M. **(b)** Cells were treated with PMA (PMA) or U73122 (U73122) and synchronized at G2/M. Nuclear accumulation of PKC⍺ and Cyclin B1 resulted linked to DAG fluctuations during cell cycle. **(c)** Cells were synchronized at G0/G1, G1/S and G2/M and the localization of different PLCs was investigated. PLCβ1 was the only one mainly localized into the nucleus. **(d)** Immunocytochemistry was performed to further study PLCβ1 localization. **(e)** PLCβ1 was silenced (PLCβ1 KD) and cells were synchronized at G2/M. A Scrambled siRNA was used as control (Scrambled). PKC⍺ and Cyclin B1 had minor nuclear accumulation in PLCβ1 knock-down conditions. **(f)** PLCβ1 (PLCβ1 KD), PKC⍺ (PKC⍺ KD) or both the molecules (PLCβ1 KD – PKC⍺ KD) were silenced. After synchronization at G2/M phase, immunoblot analyses showed that double knock-down condition had the same effects on Cyclin B1 expression than PKC⍺ silencing alone. PLCβ1 silencing did not affect Cyclin B1. **(g)** PLCβ1 was silenced and cells synchronized at G2/M. Nuclear production of IP_3_ decreased after silencing of PLCβ1. All the experiments were repeated at least three times.

In order to better understand the mechanism by which PKC⍺ and Cyclin B1 accumulate into the nucleus at G2/M checkpoint, we investigated nuclear DAG fluctuations during cell cycle progression of K562 cells. As already reported, nuclear amount of DAG peaks at G2/M, attracting PKCs to translocate into the nucleus [6, 15, 17]. Cells were synchronized at G1/S and G2/M as previously described. Total and nuclear samples were labeled with [^3^H]-glycerol and lipids were extracted. The percentage of radioactivity corresponding to DAG was analyzed (see materials and methods for the complete protocol). Accordingly with other studies [6, 15, 17], we found a slight increase in DAG levels in total samples of cells synchronized at G2/M compared to G1/S cells. On the other hand, analyzing nuclear extracts, we observed a very high increase in nuclear DAG in G2/M cells compared to G1/S cells (Figure 5A). This evidence suggested DAG as the molecule responsible for Cyclin B1 and PKC⍺ nuclear translocation. Then, in order to simulate DAG effects on PKC⍺ nuclear import, we treated the cells with PMA (100nM) for 30 minutes, as already described [41, 42]. Nuclear fractions of G2/M cells treated with PMA showed an increase of Cyclin B1 and PKC⍺ levels compared to the controls. However, as known, DAG is the product of the action of the Phospholipases C (PLC) activity, which, hydrolyzing Phosphatidylinositol 4, 5-bisphosphate (PIP_2_), produces DAG and Inositol triphosphate (IP_3_) [43–45]. Thus, in order to reduce DAG production, we treated our cells with a potent PLC inhibitor, U73122, at a final concentration of 10μM for 16 hours. Nuclear amount of PKC⍺ in G2/M synchronized cells was analyzed. We found a strong decrease of nuclear PKC⍺ in cells treated with U73122 compared to the controls. Notably, Cyclin B1 nuclear accumulation was inhibited too (Figure 5B). Taken together, these data indicated DAG as an important factor for nuclear import of PKC⍺, which, in turn, seemed to stimulate Cyclin B1 nuclear accumulation during G2/M progression.

### Nuclear PLCβ1 is responsible for DAG regulation during cell cycle progression

In this series of experiments, we screened some of the most expressed PLC isoforms in K562 cells, in order to understand which one was responsible for nuclear DAG oscillations during cell cycle. Then, we synchronized the cells at the different phases of cell cycle and we separated nuclei from cytoplasms. Interestingly, among the PLC isoforms studied, only PLCβ1 resulted mainly localized into the nucleus of K562 cells (Figure 5C-D). Next, we transiently silenced PLCβ1 (PLCβ1 KD) and, 48 hours later, we synchronized the cells at G2/M checkpoint. We used more than a single siRNA to silence PLCβ1 expression to avoid any possible off-target effect and the experiments were repeated more than three times (see materials and methods). Notably, we observed a minor accumulation of both PKC⍺ and Cyclin B1 in PLCβ1 knock-down cells compared to the controls (Scrambled) (Figure 5E). Moreover, PLCβ1 and PKC⍺ resulted to be part of the same pathway. We silenced PLCβ1 (PLCβ1 KD), PKC⍺ (PKC⍺ KD) or both the molecules (PLCβ1 KD – PKC⍺ KD). A Scrambled siRNA was used as control (Scrambled). 48 hours after the transfections, cells were synchronized at G2/M. Interestingly, double knock-down of PKC⍺ and PLCβ1 had the same effects on Cyclin B1 expression than the silencing of PKC⍺ alone. In addition, PLCβ1 silencing did not affect Cyclin B1 (Figure 5F). Finally, in order to further confirm that PLCβ1 silencing reduced its nuclear activity and, therefore, the production of DAG, we analyzed the production of IP_3_ in nuclear lysates of G2/M cells using a PLC assay previously described [46]. According to our findings, nuclear fractions characterized by PLCβ1 knock-down showed a strong decrease in IP_3_ amount compared to the controls (Figure 5G). These findings demonstrated the importance of nuclear PLCβ1 for the oscillations of DAG, related to the different cell cycle phases.

## DISCUSSION

Although the involvement of Protein Kinases C in the regulation of cell cycle progression has already been widely investigated, little has been reported about a possible connection between PKC signalling and Cyclin B1/Cdk1. In particular, even if other classes of Cyclins (D, E and A) can be modulated by different isoforms of PKCs, Cyclin B1 was not linked to any of them so far [2, 4, 12, 47]. Previous studies from our laboratory indicated important roles for PKC⍺ in cell cycle regulation. As a matter of fact, we found that PKC⍺ was able to translocate into the nuclei of murine erythroleukemia cells (MEL) at the beginning of mitosis [19]. Here, it phosphorylated Lamin B1, stimulating its disassembly and, then, the progression through G2/M phase. More recently, we demonstrated that lipid signalling could be involved in the regulation of Cyclin D3 and, in turn, of cell proliferation in human erythroleukemia cells, K562. Indeed, we observed that overexpression of PLCβ1 drove to an up-modulation of Cyclin D3 and a down-regulation of PKC⍺ expression [12]. This was followed by an accumulation of cells at G1/S transition. In particular, silencing of PKC⍺ led to a very similar up-regulation of Cyclin D3 compared to PLCβ1 overexpression [12]. Taken together, these evidences suggested an important role for PKC⍺ in cell cycle modulation of K562 cell line. Moreover, as up-modulation of D-type cyclins is commonly linked to down-regulation of the B-type isoforms, we decided to understand if PKC⍺ could be also involved in G2/M progression targeting Cyclin B1 [21]. In this study, we described a novel and peculiar DAG-dependent mechanism through which PKC⍺ regulates Cyclin B1, leading to effects on G2/M transition. Indeed, using different PKC inhibitors, we observed a strong decrease of Cyclin B1 expression consequent to PKCs down-regulation. Notably, inhibiting only the activity of PKCs, we did not find any effect on Cyclin B1 expression. As the effects of two of the inhibitors that we used, Go6976 and Go6983, have already been widely described in literature [33], surprising was the capacity of the PKC inhibitor to downregulate the levels of conventional PKCs in our cell model (at 1μM it seemed not specific only for PKCβ, see materials and methods). As the gene expression of the enzymes was not targeted, this peculiar action on PKCs was probably due to side effects on other proteins involved in PKC life cycle. However, this mechanism must be further studied in the future. Moreover, treating the cells with PMA, in order to stimulate the PKC signalling, we obtained, a strong decrease in PKC expression, followed by a drop of Cyclin B1 levels. If PKC decrease was related to their iper-activation by PMA, as explained elsewhere [35–37], Cyclin B1 down-regulation appeared to be related to the amount of PKCs present in the cells. In particular, to avoid any oscillation of Cyclin B1 expression, all the experiments were performed synchronizing the cells at the same cell cycle phase. As K562 cell line is characterized by the presence of only two DAG-dependent PKC isoforms, PKC⍺ and PKCβII [12, 36], we focused on these two molecules. Experiments of RNA interference showed that only in PKC⍺ knock-down conditions the levels of Cyclin B1 drop in the cells. This led to an accumulation of the cells at G2/M, which could be related to the lack of Cyclin B1 caused by PKC⍺ silencing. Indeed, accordingly to literature, a high decrease of Cyclin B1 could interfere with the transition through the G2/M phase of cell cycle [38]. In addition, this mechanism resulted independent by the kinase activity of PKC⍺. Overexpression of a Dominant Negative (DN) mutant of PKC⍺ drove to the same up-modulation of Cyclin B1 and cell cycle progression than the Wild Type isoform (WT). Next, investigating this regulatory mechanism, we found that PKC⍺ did not regulate the gene expression of Cyclin B1, but it affected its degradation. Notably, experiments performed using the proteasome inhibitor MG-132 indicated that Cyclin B1 degradation raised with lack of PKC⍺. Our findings were supported by p21 up-modulation during PKC⍺ silencing. Indeed, the expression of this Cip/Kip inhibitor has already been described to increase during Cyclin B1 degradation [40]. Next, we decided to investigate the behavior of these two molecules during cell cycle progression. They resulted able to co-localize during the different cell cycle phases, accumulating into the nucleus, especially at G2/M. Next, experiments of co-immunoprecipitation indicated the possibility for the two proteins to interact. In addition, as further demonstration of that, PKC⍺ resulted able to immunoprecipitated also with cdk1/cdc2, the kinase which interacts with Cyclin B1 and regulates G2/M progression of the cell cycle [20–28]. In particular, the complex appeared to be located in the cytoplasmic fraction and to take place along the cell cycle. These evidences suggested a possible role for PKC⍺ in preserving Cyclin B1 from degradation thanks to its physical interaction with it. Moreover, PKC⍺ nuclear translocation during mitosis seemed to modulate Cyclin B1 import. Indeed, silencing or overexpression of PKC⍺ led to an important modulation of the amount of nuclear Cyclin B1. Then, we decided to understand which was the factor that promoted PKC⍺ nuclear import during mitosis. We observed that nuclear DAG amount, as already reported in other models [14–18], raised in the nucleus of K562 cells at G2/M. Considering it as the possible trigger for PKC⍺ and Cyclin B1 nuclear import, we decided to simulate its accumulation using PMA, as already described by other studies [41, 42]. This treatment increased the accumulation of both PKC⍺ and Cyclin B1 in the nuclei of the cells. On the contrary, in order to limit DAG production, as Phospholipases C (PLC) are a family of proteins able to hydrolyze Phosphatidylinositol 4,5-bisphosphate (PIP_2_), producing DAG and IP_3_ [44–46], we inhibited their activity treating the cells with a PLC inhibitor, U73122. Interestingly, we found a minor nuclear accumulation of the two proteins. Next, we investigated which PLC isoform could be responsible for DAG production during cell cycle progression. Notably, we noticed that the only isoform mainly localized in the nuclei was PLCβ1, capable to modulate nuclear import of PKC⍺ and Cyclin B1 during mitosis. Indeed, silencing this isozyme led to the same minor accumulation in the nuclei of the two molecules observed using the U73122 inhibitor. In particular, Cyclin B1 resulted to be downstream of the PLCβ1/PKC⍺ pathway. Indeed, experiments of double knock-down of the two molecules did not have any higher effect on Cyclin B1 expression than the PKC⍺ silencing alone. Finally, we proved that PLCβ1 silencing inhibited the production of IP_3_ in the nucleus of G2/M cells. These results indicated that the lipase was responsible of lipid changes during cell cycle progression. What emerges by our study is a novel mechanism, through which PKC⍺ results fundamental for stability and nuclear import of Cyclin B1 during G2/M progression. This process is mediated by cell cycle related nuclear DAG fluctuations, which are due to the activity of nuclear PLCβ1.

## MATERIALS AND METHODS

### Cell culture

Human erythroleukemia cells (K562) were grown in RPMI 1640 (Sigma Aldrich) supplemented with 10% heat-inactivated fetal bovine serum (FBS) and L-glutamine/streptomycin (1×). Cells were treated with the following compounds: Phorbol 12-myristate 13-acetate (PMA, Sigma Aldrich) at a final concentration of 50-100nM for 30 mins or 16 hours, U73122 (Sigma Aldrich, PLC inhibitor) at 10 μM for 16 hours, with Go6976 and Go6983 at 1μM (Sigma Aldrich, PKC inhibitor) and with 3-(1-(3-imidazol-1-ylpropyl)-1H-indol-3-yl)-4-anilino-1H-pyrrole-2,5-dione anilinomonoindolylmaleimide (Calbiochem, PKC inhibitor [34]) at 1 μM for 24 hours. Finally, MG-132 (Sigma Aldrich, proteasome inhibitor) was used at a final concentration of 15 μM for 2 hours.

### Cell synchronization

Cells were collected as described below for the experiments: (1) G1/S cells: proliferating cells were cultured in complete RPMI 1640 FBS 10%; (2) G2/M blocked cells: growing cells were treated with Nocodazole (Sigma Aldrich) at a final concentration of 20 ng/ml for 16 h; (3) G0/G1 cells: growing cells were starved in Hank's Balanced Salt Solution (HBSS, Sigma Aldrich) for 16 hours. In experiments of PKC⍺ overexpression, cells were partially stimulated to undergo G2/M accumulation adding Nocodazole for 6 hours at a final concentration of 20ng/ml

### Western blot analysis

Cells were collected by centrifugation, washed in PBS and lysed in mPER lysis buffer (BIORAD) containing protease inhibitors (Thema Ricerca). Samples were separated on SDS-PAGE and electro-transferred to nitrocellulose membranes. Membranes were washed in PBS-0.1%Tween-20 (PBS/T) and nonspecific binding sites were blocked by incubation in blocking buffer (PBS/T with 5% w/v non-fat dry milk) for 1 h at room temperature. After several PBS/T-washes, membranes were incubated with primary antibodies overnight at 4°C. Membranes were washed again, then incubated with peroxidase conjugated secondary antibodies diluted in PBS/T for 1 h at room temperature. Proteins were detected by incubating membranes in enhanced chemiluminescence detection system (ECL, Thema Ricerca). Antibodies were as follows: PLCβ1, Cyclin D3, Cyclin B1, PKC⍺, PKCβII, Lamin A/C, from Santa Cruz, PKC⍺ and Cyclin B1, phospho-Cyclin B1 Ser133, phospho-Cyclin B1 Ser147, Cdc25c, Cdk1/cdc2, phospho-Cdk1/cdc2 Tyr15, Cdk7 from Cell Signaling technology and β-tubulin from Sigma Aldrich. Analysis with an antibody for β-tubulin demonstrated equal protein loading.

### Flow cytometric analysis of cell cycle

For FACS analysis 1 × 10^6^ cells were collected by centrifugation at 1,200 rpm for 5 min at 4°C and washed twice in ice-cold PBS. Cells were fixed (and permeabilized) with −20°C cold 70% ethanol overnight at 4°C. Fixed cells were, then, washed in PBS twice and resuspended in 1 ml of staining solution (40 μg/ml propidium iodide and 100 μg/ml RNase A in PBS). The samples were incubated for at least 30 min at room temperature in the dark. FACS analysis was performed, and the percentage of cells in different phases of the cell cycle was assessed using a FC500 flow cytometer equipped with cxp software (Beckman Coulter Inc.). At least 10,000 events per sample were acquired.

### Cell transfections

Cells were transfected with full-length DNA vectors for human PKC⍺ (Addgene, plasmid number 21232 and 21235) [48] using empty pcDNA/2.1 plasmid (Invitrogen) as control. Overexpressions were performed using Lipofectamine 2000 from Invitrogen. Cells were seeded at a cell density of 5 × 10^5^/ml in 6-well plates, to which was added the mix of Lipofectamine 2000 (Life Technologies) and right vectors, following manufacturer's instructions. The expression of PKC⍺, PKCβII and PLCβ1 was silenced by RNAi at a final concentration of 50nM using the electroporation assay kit by Thema Ricerca (program T-16) and Lipofectamine RNAiMAX (Life Technologies) as indicated by the manufacturers' instructions. The following siRNAs were used: in order to silence PLCβ1 s23358 and s23359 (Applied Biosystems), to silence PKC⍺ s11092, s11093 and s11094 (Applied Biosystems), to silence PKCβII s11095 (Applied Biosystems). As negative control a mix of Silencer Select Negative Control #1 and #2 siRNAs (Applied Biosystems).

### Nuclear extraction and purification

Cells were collected, centrifuged and washed in PBS 1X. PBS 1X was accurately removed, and the pellets were resuspended in 1ml of TM2 buffer (10 mM Tris-HCl pH 7.4, 2 mM MgCl_2_) for 2 minutes. Then, Triton 0.6% was added and everything was passed twice through a syringe with a 22 ½ gauge needle. Next, MgCl2 3mM was added to the solution, which was centrifuged for 10 mins at 0.8 rpm. The nuclear pellets were washed twice in TM5 buffer (10 mM Tris-HCl pH 7.4, 5 mM MgCl_2_), while the supernatant was transferred in a new vial and used like a cytoplasmic control.

### Immunoprecipitation

Cells were lysed in mPER lysis buffer (BIORAD) containing protease inhibitors (Thema Ricerca). 500 μg of proteins were pre-cleared adding 20μl of Protein A/G PLUS-Agarose (Santa Cruz) for 1 hour at 4°C. Then, beads were centrifuged and the supernatant transferred in another vial. 500 μl of protein lysates were incubated over/night with the appropriate primary antibodies on wheels at 4°C. The day after, 25 μl of Protein A/G PLUS-Agarose were added for 1h at 4°C. Beads were centrifuged and pellets washed 3 times with mPER. Immuno-complexes were resuspended in loading buffer (4X), boiled at 95°C for 5 minutes, resolved on SDS/PAGE and transferred on nitrocellulose. After incubation with the indicated antibodies, antibody–protein interactions were detected with enhanced chemiluminescence detection system (ECL, Thema Ricerca). As positive control 50 μg of proteins from total lysates of K562 were loaded on SDS/PAGE gel.

### Immunofluorescence microscopy

Cells were seeded on electrostatically charged glass slides using a Shandon Cytospin (Thermo Electron Corporation, Pittsburgh, PA, USA) at low acceleration and 200 rpm for 5 min. Slides were fixed in 4% paraformaldehyde at 37°C for 10 min and permeabilized with 0.15% Triton X-100 in PBS for 8 min. Then slides were blocked with PBS containing 5% BSA for 1 h. Incubation with monoclonal anti-PKC⍺ (1:100) (Santa Cruz) and Cyclin B1 (1:100) (Santa Cruz) was performed overnight at 4°C in blocking medium and then with FITC-conjugated anti-mouse immunoglobulin G (IgG) antibody (1:200) or Cy3-conjugated anti-rabbit IgG antibody (1:100) (Sigma-Aldrich) for 1 h at room temperature. Slides were washed 3 times for 10 min at room temperature with PBS/Tween and mounted with a DAPI anti-fade reagent in glycerol (Molecular Probes, Eugene, OR, USA). Images were taken on a Zeiss Axio ImagerZ1 microscope, equipped with 60X/NA 1.4 optics and Apotome apparatus, coupled to a computer driven Zeiss AxioCam digital camera (MRm), using Zeiss Axio Vision 4.4 software (Carl Zeiss, Oberkochen, Germany).

### RNA extraction, retrotranscription and real-time PCR analysis

RNA extraction, retrotranscription and real-time PCR were performed as already described [12]. Gene expression were assessed using for PKC⍺ the probe Hs00925193_m1, for Cyclin B1 the Hs00820463_g1 and for PLCβ1 the Hs00248563_m1 (Applied Biosystems).

### Cell Counting

To determine cell growth, cells were synchronized using Nocodazole and, 24 hours later, the block was removed seeding them in complete RPMI 10% FBS at a cell density of 1 × 10^5^/ml in 6-well plates. Growth curves were determined by direct counting of cells harvested for 24, 48 and 72 h after seeding. Viable cells were handly counted by a hemocytometer using 0.2% Trypan Blue.

### Statistical analysis

Statistical analyses were performed by Student t-test, using GraphPad Prism (GraphPad Software Inc. version.6) (*P < 0.05, **P < 0.001, ***P < 0.0001).

### Quantification of DAG amount

K562 cells were cultured and synchronized as previously described. Nuclei or intact cells were labeled with [^3^H]-glycerol (10 μCi/ml/1 × 10^6^ cells) for 90 minutes. Next, nuclei and total cells were precipitated with 10% TCA and, then, 10 volumes of chloroform/methanol/concentrated HCl (300:300:1.5) were added and lipids extracted for 20 hours at 4°C. After centrifugation, supernatants were preserved and the pellets were re-extracted twice with 10 volumes of chloroform/methanol/concentrated HCl (400:200/1.5). The combined supernatants were dried under steam of nitrogen and lipids were dissolved in 100ul of chloroform and washed three times in 4 volumes of chloroform/methanol/water (3:48:47). Finally the samples were dried under a steam of nitrogen and [^3^H]-labelled lipids were analyzed by TLC on silica gel 60 plates developed with ether/exane/NH4 (50:50:0.25). TLC plates were sprayed with Enhancer (Du Pont, NEN) and fluorographed at −80°C. Spots corresponding to lipids were scraped off, extracted with 1.5 ml of 0.6N HCl-Methanol (60:40 by volume) for 48 hours with gentle stirring and counted with a liquid scintillation counter using 9ml of Packard Pico-Fluor 40 scintillation cocktail.

### PLC assay

The activity of nuclear PLCβ1 was assessed using a protocol described elsewhere [46].

## SUPPLEMENTARY FIGURES



## ABBREVIATIONS

PKC: Protein Kinase C
PLC: Phospholipase C
DAG: diacylglycerol
IP_3_: inositol 1,4,5-trisphosphate
Ca^2+^: Calcium
Cdk: cyclin-dependent kinase
CRS: cytoplasmic retention signal
NLS: nuclear localization signal
PMA: phorbol-12-myristate-13-acetate.
