# Supplementary material for: A novel DAG-dependent mechanism links PKCa and Cyclin B1 regulating cell cycle progression
Source: Oncotarget. 2014 Oct 24;5(22):11526–40. doi: 10.18632/oncotarget.2578 (PMC4294327; doi:10.18632/oncotarget.2578)
Supplement: Supplementary file 1 [file oncotarget-05-11526-s001.pdf]

SUPPLEMENTARY FIGURES

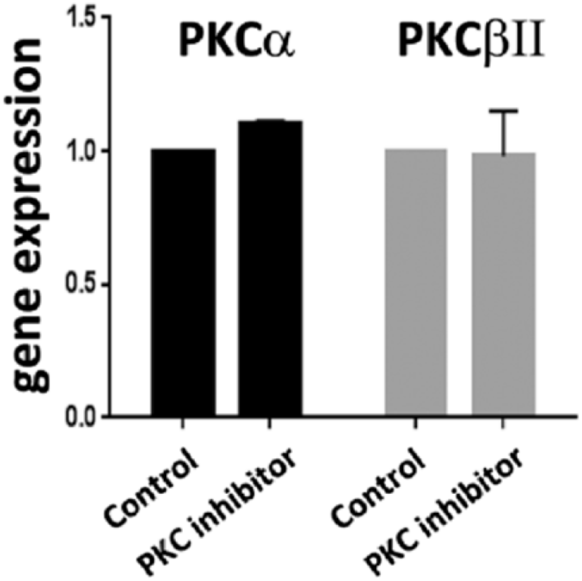

Supplementary Figure 1: PKC inhibitor does not downregulate the gene expression of conventional PKC isoforms expressed in K562 cell line. Cells were treated with PKC inhibitor as explained in materials and methods and gene expression analyses were performed via qPCR. The gene expression of PKCα and PKCβII was not affected by the action of PKC inhibitor.

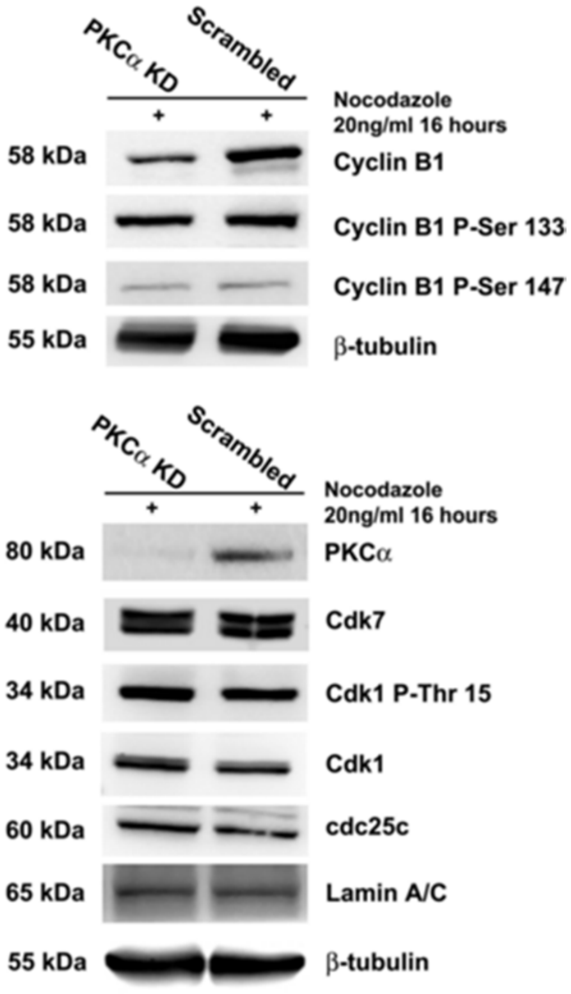

Supplementary Figure 2: PKCα silencing had no effects on Cyclin B1 phosphorylations or G2/M related proteins. **(a)** PKCα was silenced (PKCα KD) and cells synchronized at G2/M using Nocodazole. A Scrambled siRNA was used as control (Scrambled). Immunoblot analyses were performed to investigate phosphorylations of Ser-133 and Ser-147 on Cyclin B1. No changes in their levels were detected. **(b)** PKCα was silenced and cells synchronized as a). Immunoblot analyses were performed to investigate the expression of several proteins related to G2/M phase. No changes in their levels were detected.

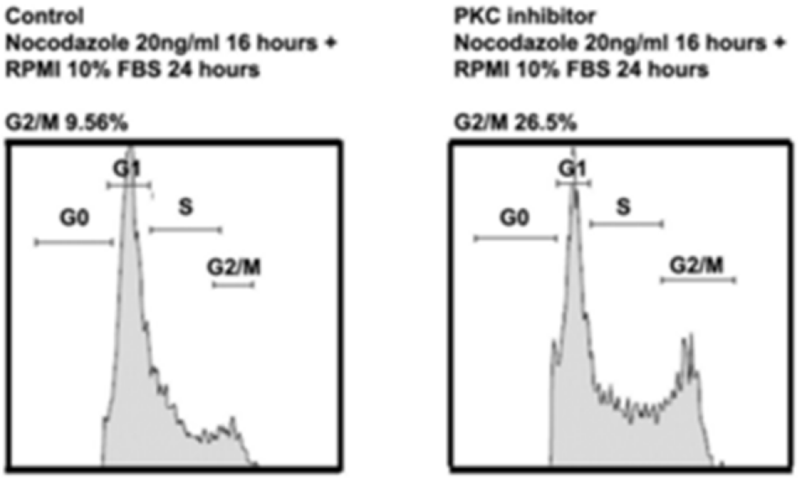

Supplementary Figure 3: Cells treated with PKC inhibitor showed accumulation at G2/M checkpoint a) Cells were treated with PKC inhibitor and synchronized at G2/M using Nocodazole. Then, G2/M block was removed seeding the cells in complete RPMI 10 % FBS for 24 hours. PKC inhibitor led to higher accumulation at G2/M compared to the control.

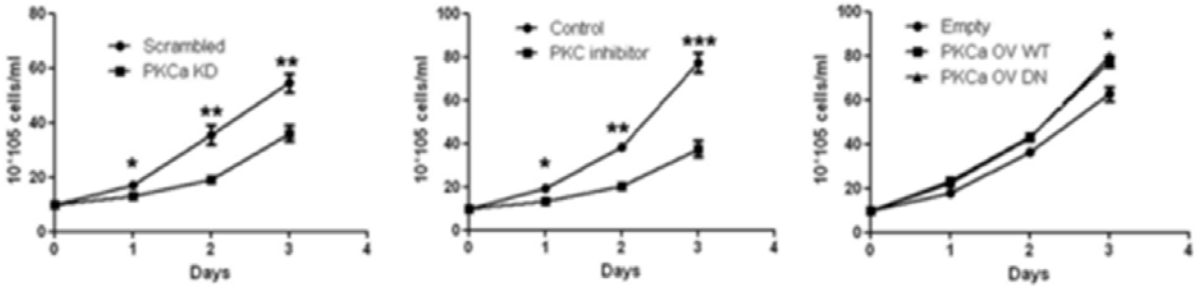

Supplementary Figure 4: PKC silencing or PKC inhibitor treatment slowed-down cell proliferation of K562 cell line if compared to the controls. On the contrary, PKCα overexpression (WT/DN) accelerated it. Cells were transfected to silence or overexpress PKCα or treated with PKC inhibitor for 24 hours. Next, they were synchronized at G2/M using Nocodazole. Finally, the G2/M block was removed and the cells were seeded in complete RPMI 10% FBS at a cell density of 10 × 105/ml and counted for 72 hours. Cell countings were performed in triplicate and analyzed by Student's t-test (*$p < 0.05$).

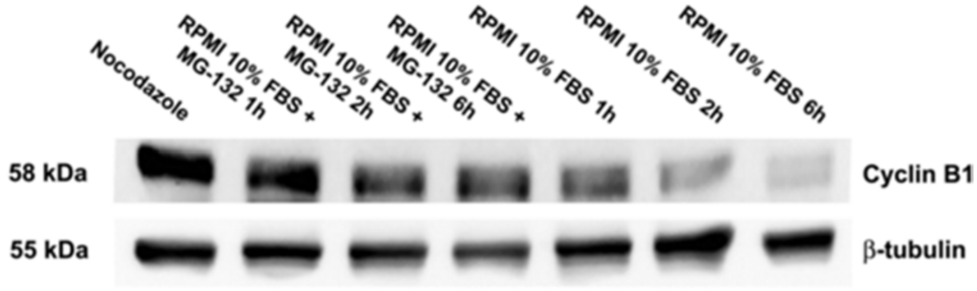

Supplementary Figure 5: Cyclin B1 degradation after G2/M block release. Two aliquots of cells were synchronized at G2/M using Nocodazole and, 16 hours later, seeded again in complete RPMI 10% FBS. One aliquot was treated with MG-132 for 1/2/6 hours and one was used as control. Cyclin B1 degradation was detected through immunoblot analysis.

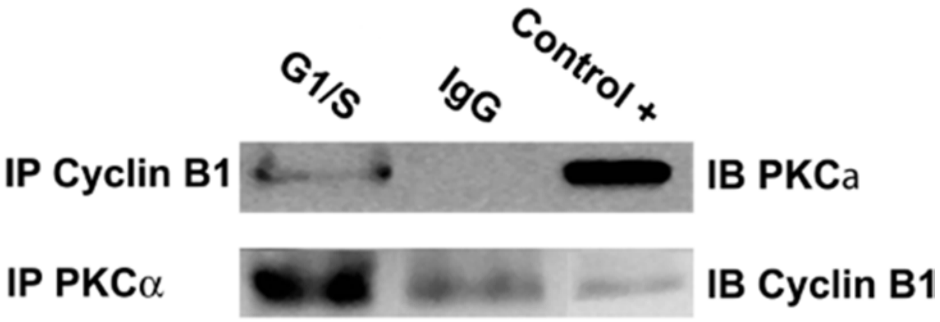

Supplementary Figure 6: PKCα and Cyclin B1 interaction is detectable also in G1/S cycling cells (G1/S). Co-immunoprecipitation experiments were performed in cycling cells. As negative control non-specific IgG were used (IgG). 50μg total lysate of K562 were used as positive control (Control +).

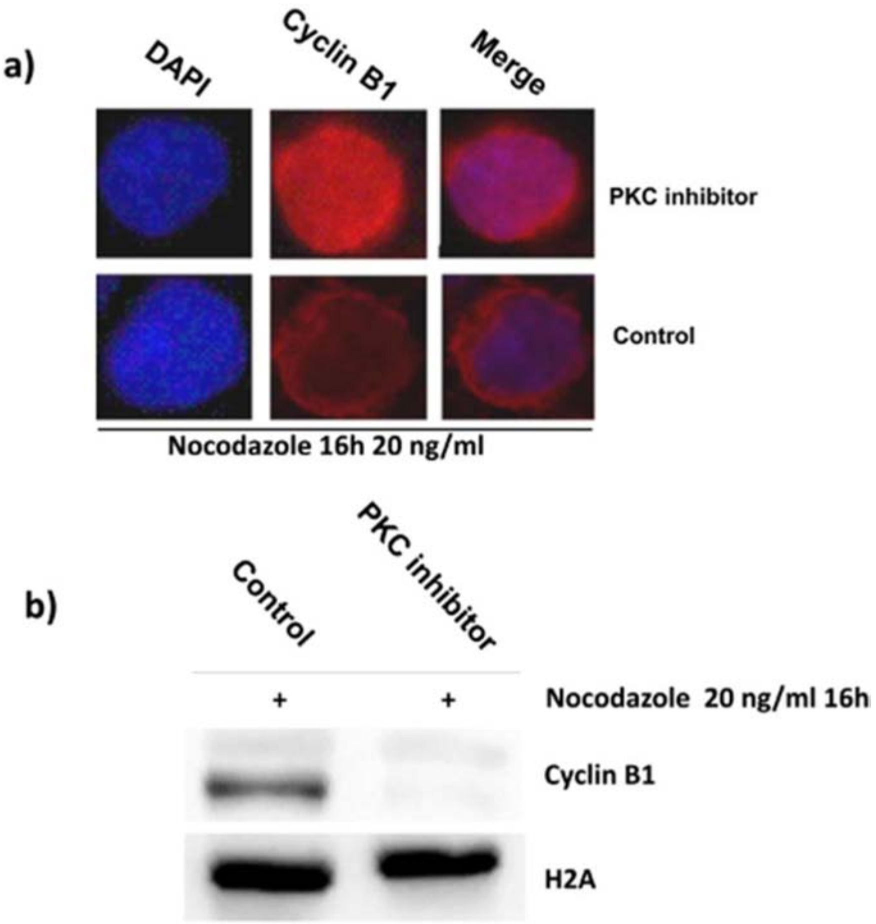

Supplementary Figure 7: PKC inhibitor decreased the nuclear accumulation of Cyclin B1 at G2/M checkpoint. **(a)** Cells were treated with PKC inhibitor and synchronized at G2/M using Nocodazole. Immunocytochemistry analysis was performed to study Cyclin B1 nuclear accumulation, which resulted minor in cells treated with PKC inhibitor respect to the control. **(b)** Cells were treated as a) and Immunoblotting analysis of nuclear lysates was performed. Cells treated with PKC inhibitor (PKC inhibitor) showed a minor amount of Cyclin B1 compared to the control (Control).
